# The Value of Fetal Heart Evaluation in Fetuses with Rare Congenital Lymphangiomas: A Cohort Study from a Single Tertiary Center across Two Decades (Years 1999–2020)

**DOI:** 10.3390/jcm11041035

**Published:** 2022-02-16

**Authors:** Paulina Kordjalik, Bartosz Szmyd, Filip Franciszek Karuga, Gabriela Daszkiewicz, Iwona Strzelecka, Maria Respondek-Liberska

**Affiliations:** 1 Department of Prenatal Cardiology, Polish Mother’s Memorial Hospital, 93-338 Łódź, Poland; pkordjalik@gmail.com (P.K.); maria.respondek-liberska@uni.lodz.pl (M.R.-L.); 2 Department of Pediatrics, Oncology, and Hematology, Medical University of Lodz, 91-738 Łódź, Poland; bartoszmyd@gmail.com; 3 Department of Sleep Medicine and Metabolic Disorders, Medical University of Lodz, 92-215 Lodz, Poland; filip.karuga@umed.lodz.pl; 4 Student’s Scientific Association Prenatal Cardiology, Medical University of Lodz, 93-338 Łódź, Poland; gabriela.daszkiewicz@icloud.com; 5 Department of Diagnosis and Prevention of Fetal Malformations, Medical University of Lodz, 93-338 Łódź, Poland

**Keywords:** echocardiography, lymphangioma, prenatal cardiology, tumor, ultrasonography

## Abstract

Lymphangiomas are uncommon, benign (from a histopathology viewpoint) malformations of the lymphatic system with thin-walled vessels; however, these tumors may be dangerous for fetal or neonatal life. They are observed in 1:6000 newborns at birth and in 1:750 spontaneous abortions. We aimed to investigate the role of fetal echocardiography in the prognosis of lymphangioma. Selected data of 19,836 pregnant women studied between 1999 and 2020 were retrospectively analyzed. In total, 32 cases of lymphangioma meeting the following criteria were further analyzed: (1) ultrasound availability from the 1st trimester of pregnancy and (2) nuchal translucency ≤ 2.5 mm. Echocardiographic findings, karyotype, size, and location of the possible lesion were juxtaposed with the clinical follow-up. The statistical analysis was performed using Statistica 13.1 software (StatSoft, Tulsa, OK, USA). Lymphangioma in the analyzed material coexisted with abnormalities in fetal echo in 78% (*n* = 25) of cases, especially: heart defect in 50% (*n* = 16) and with normal heart structure with functional changes in 28% (*n* = 9). Karyotype was available in 50% of the analyzed cases (*n* = 16). Normal cytogenetic results were observed in 62.5% (*n* = 10) of cases. In the remaining cases, the following were observed: Turner Syndrome: 25% (*n* = 4) and Down Syndrome 12.5% (*n* = 2). The rate of alive newborns was significantly higher among fetuses with isolated lymphangioma in comparison to those with lymphangioma associated with abnormal ECHO examination: 38.46% (*n* = 5) vs. 15.38% (*n* = 2; *p* = 0.037). Abnormal ECHO exam was a poor prognostic sign for fetuses with lymphangioma; therefore, we think it is important to refer these cases for detailed echocardiography in tertiary centers. Moreover, it should be highlighted that in all lymphangioma cases there was an indication to perform the karyotype assessment, as there was a high risk of aneuploidy.

## 1. Introduction

Lymphangiomas are uncommon, benign (from a histopathology viewpoint) malformations of the lymphatic system with thin-walled vessels; however, these tumors may be dangerous for fetal or neonatal life [1,2]. They are observed in 1:6000 newborns at birth and in 1:750 spontaneous abortions. In the majority of cases, they involve the neck or axilla, but can also involve other parts of the body, such as: scrotum, retroperitoneum, gluteal region, mediastinum, groin, pelvis, mesentery, omentum and spleen [3,4,5,6,7,8].

Their detection in fetal life is possible due to common prenatal ultrasound examinations; however, diagnosis and prognosis should be established without histopathology evaluation. To date, there has not been a comprehensive study on the importance of echocardiography in assessing the prognosis of a fetus with lymphangioma. The differences in the prognosis of fetuses with isolated lymphangioma in comparison to fetuses with lymphangioma coexisting with heart defects are also unknown. It is all the more important to delve into this topic, as the risk factors in fetuses with a normal karyotype are unknown.

Thus, we investigated the role of fetal echocardiography in the definition of prognosis of lymphangioma by comparing the outcomes in fetuses with isolated lymphangioma and associated with abnormal heart anatomy/study.

## 2. Materials and Methods

### 2.1. General Part

Selected data of 19,836 pregnant women examined in the Department of Prenatal Cardiology of Polish Mother’s Memorial Hospital Research Institute from 1999–2020 were retrospectively analyzed. In our study, we proposed a modified definition of lymphangioma as a multiseptated, multicystic irregular mass in a subgroup of fetuses who exhibited normal NT in the 1st trimester of pregnancy (<2.5 mm) [9]. This modification of the currently used definition is due to the fact that, from our point of view, cystic hygroma in the 1st trimester is not the same disease—although, in some cases, it may persist in the 2nd trimester and even progress and show very similar presentation as typical lymphangioma. In the results, we found 32 cases of lymphangioma that fulfilled the following criteria, which were further analyzed: 1) ultrasound availability from the 1st trimester of pregnancy and 2) nuchal translucency ≤ 2.5 mm. Data from prenatal screening (pregnant women’s age, place of residence, echocardiographic findings (e.g., extracardiac abnormalities (ECA) and/or malformations (ECM) such as: polyhydramnios, oligohydramnios, pyelectasis bilateralis, non-immune hydrops fetalis, skin oedema, ascites), karyotype, size, and location of any lesion) were analyzed and juxtaposed with the clinical follow-up (used treatment option, treatment effects, survival). The study was conducted according to the guidelines of the Declaration of Helsinki. All patients consented to the use of data for the scientific analysis after its anonymization (this agreement is routinely obtained from all patients diagnosed in our department). Ethical review and approval were waived for this study due to its retrospective character and the fact that it only involved data routinely obtained during prenatal cardiology examination accepted earlier by the Institutional Board and Bioethics Committee.

### 2.2. Statistical Analysis

Continuous variables were presented as average ± standard deviation, normal distribution and, otherwise, as median with interquartile range (IQR: quartile 1–quartile 3). The normality of obtained data was assessed using the Shapiro–Wilk test. Nominal variables are presented as numbers with the percentage of the total. The statistical analysis was performed using Statistica 13.1 software (StatSoft, Tulsa, OK, USA). Moreover, the Fisher exact test for a stronger association was calculated according to the definition [10].

## 3. Results

The median age of pregnant women of the analyzed group was 28 years (IQR: 24.75–31.25). The median gestational age of detected anomalies in the analyzed group according to the date of last menstrual period (LMP) was 18 weeks (IQR: 14–22), according to biometry 18 (IQR: 15–22).

**Figure 1:**
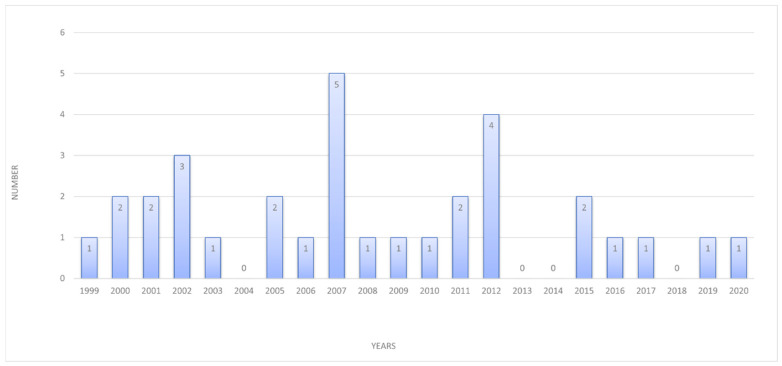
Number of fetuses with lymphangioma from 1999–2020 in Department of Prenatal Cardiology Polish Mother’s Memorial Hospital Research Institute.

Between 1999 and 2020, a single case (IQR: 1–2) of lymphangioma was diagnosed per year. The highest number of defects was detected in 2007—five cases (χ2 with Yate’s correction; *p* < 0.005; see Figure 1). Lymphangioma in analyzed material coexisted with:

- heart defect in 50% (*n* = 16);
- normal heart structure with functional changes in 28% (*n* = 9), including:
  ○ tricuspid regurgitation in 22% (*n* = 7);
  ○ fetal rhythm disturbances in 6% (*n* = 2).

**Figure 2:**
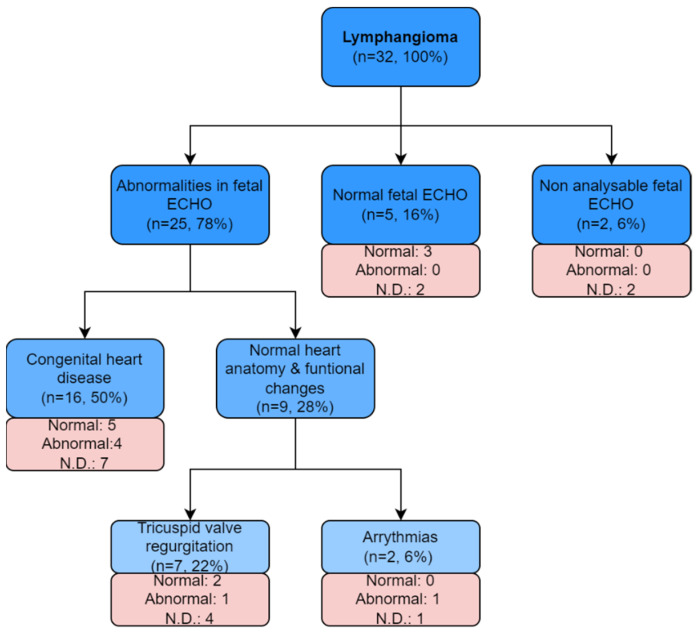
Abnormalities in echocardiography examination among fetuses with lymphangioma (blue boxes). Pink boxes show the karyotypes in each subgroup. Legend: N.D.—no data available.

In 6% (*n* = 2) of cases, echocardiography was so technically difficult due to maternal obesity and an obscuring tumor lesion that no interpretable fetal heart images were obtained (see Figure 2).

**Figure 3:**
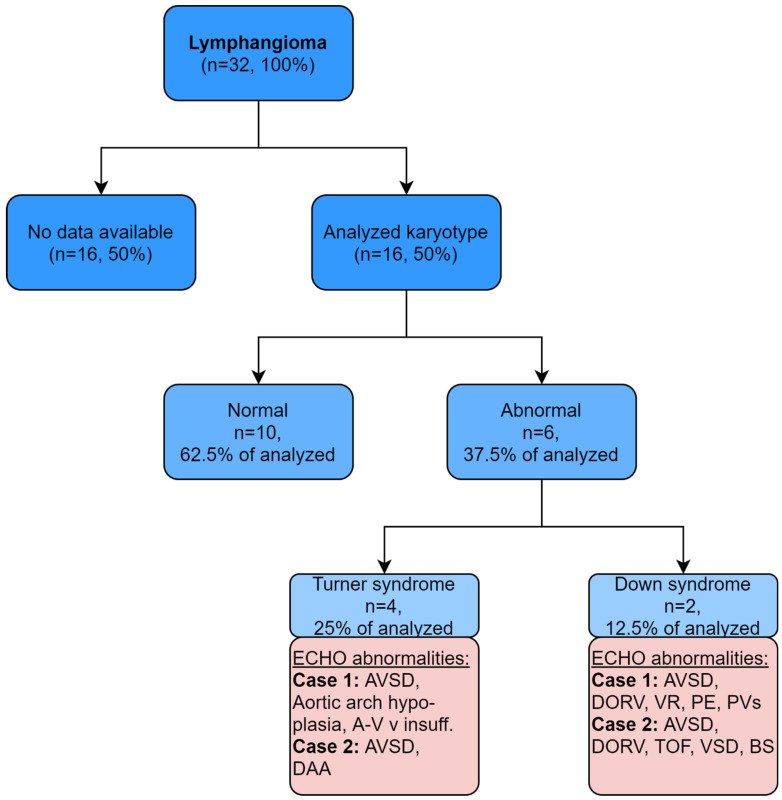
Karyotypes and related ECHO abnormalities among fetuses with lymphangioma. Legend: A-V v insuff—atrioventricular valve insufficiency; AVSD—atrioventricular septal defect; BS—bright spot; DAA—double aortic arch; DORV—double outlet right ventricle; PE—Pericardial effusion; PVs—pulmonary valve stenosis; TOF—tetralogy of Fallot; VSD—ventricular septal defect.

In the available material, genetic testing was limited only to cytogenetic analysis, available in 50% of the analyzed cases (*n* = 16). The karyotype in fetuses with lymphangioma revealed a normal cytogenetic result in 62.5% (*n* = 10) of cases. In the remaining cases, the following were observed: Turner Syndrome: 25% (*n* = 4) and Down syndrome 12.5% (*n* = 2) (see Figure 3). In four cases, abnormal karyotype was associated with abnormal echocardiography (see Figure 3, pink part).

**Figure 4:**
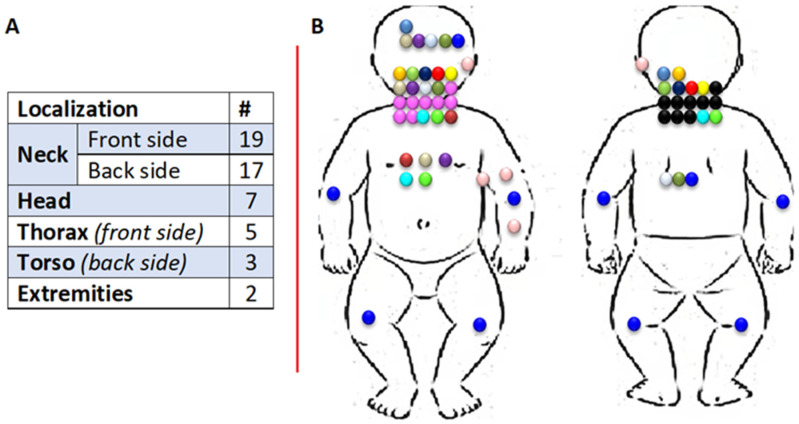
Areas affected by lesions of the lymphangioma type in the fetus in tabular (**A**) and graphical (**B**) version. Legend: if one color of dot is observed in multiple localization, it represents one fetus with multiple lymphangiomas; more than one color in one localization represents the number of single lesions in this place.

Data on body regions of fetuses occupied by lymphangiomas were further analyzed. They involved a single body part or several body regions (see Figure 4). The neck was the most affected area at 59% (*n* = 19)—front side and 53% (*n* = 17)—back side, followed by the head at 22% (*n* = 7). Performed statistical analysis did not reveal the impact of lymphangioma localization on the follow-up, what may be an effect of a relatively small number of lymhangioma patients (see Supplementary Materials for further information).

Termination of pregnancy occurred in 18% (*n* = 6), in utero death was reported in 44% (*n* = 14), neonatal death was reported in 16% (*n* = 5), and 22% (*n* = 7) of neonates survived (see Table 1). The histopathological examination was performed in one case of neonatal death (46, XY CHD and neck lymphangioma) and revealed: ascites, hydrothorax, generalized oedema of subcutaneous tissue (up to 3 cm), myocarditis, lungs hypoplasia, hepatomegaly, spleen lesions, right kidney agenesis, infections’ symptoms. In three other cases, histopathological examinations confirmed prenatal diagnosis. The follow-up of fetuses with lymphangioma and normal heart structure was compared with that of fetuses with lymphangioma and heart defect (*n* = 16). The Fisher exact test for a stronger association revealed that the rate of alive newborns was significantly higher among fetuses with isolated lymphangioma in comparison to these with lymphangioma associated with abnormal ECHO examination: 38.46% (*n* = 5) vs. 15.38% (*n* = 2; *p* = 0.037)—without termination of pregnancy and 31.25% (*n* = 5) vs. 12.5% (*n* = 2; *p* = 0.041) including these cases.

In addition, the place of residence of 22 pregnant women whose fetuses were diagnosed with lymphangioma was analyzed; in 10 cases, the pregnant women did not provide a permanent residence. The largest number of fetuses with lymphangioma came from the Kuyavian-Pomeranian *n* = 6, Łódzkie *n* = 6, and Lower Silesian *n* = 3 voivodeships. One case each was reported from Lubuskie, Wielkopolskie, Mazovian, Subcarpathian, Pomeranian, and Świętokrzyskie voivodeships.

### Follow-Up

Follow-up showed that neonatal period survival rate was 22% (7 neonates with lymphangioma: 2 cases were associated with congenital heart disease (1. Ebstein syndrome + functional pulmonary valve atresia, 2. Down syndrome + atrioventricular septal defect, double outlet right ventricle, pulmonary valve stenosis, pericardial effusion)). They were 50% natural-born infants (the caesarean section was indicated in extensive lymphangiomas and lymphangioma + CHD); the average birth weight of newborns ranged from 1700 to 4380g; in one case, surgical intervention was necessary—the injection of the lymphatic lesion with Picibalin, whereas, in the remaining seven cases, lesions regressed. The longest neonatal hospital stay lasted 54 days due to cardiosurgical intervention for heart defects—atrioventricular septal defect (AVSD), double outlet right ventricle (DORV) and pulmonary valve stenosis (PvS).

## 4. Discussion

In the Polish health care system, prenatal ultrasound is usually performed four times during pregnancy: before 10 (to confirm the pregnancy), between 11–13 (1 trimester with nuchal translucency measurement), between 18–22 (anomaly scan), and 28–32 weeks of fetal life (fetal growth assessment) [11].This provides the opportunity for early detection of many changes, including hamartomas of the lymphatic vessels called lymphangiomas [12,13]. In our unit, which is a referral center for fetal malformations, we recognized 32 cases of lymphangioma among 19,836 fetuses from high-risk pregnancies examined in the tertiary center for fetal malformations between the years of 1999–2020. The rate is higher than what can be found in data from the literature, which stated that this lesion is observed among 1:6000 neonates at birth and 1:750 in spontaneous abortions, in an average low-risk population [2]. This may be explained by the fact that we take into consideration all observed prenatally cases, as well as those that ended with death in utero and termination of pregnancy (> 60% cases). Taking this into account, these ratios are at a comparable level, which may prove the representativeness of the group. Jiao-Ling et al.’s study diagnosed only 79 cases of lymphangioma out of more than 133,000 fetuses studied [14]. Some authors have reported a much higher prevalence of this type of fetal lesion on ultrasound [15]. This is most likely due to the inclusion of cystic hygroma and lymphangioma lesions as the same anomaly. In our unit, we distinguish between these terms—cystic hygroma is referred to in the first trimester of pregnancy and may progress later on or may regress. By our definition, lymphangioma is not present in the 1st trimester but manifests in the second half of pregnancy and is usually characterized by progressive growth. The median week of lymphangioma detection in our series was 18 weeks of gestation according to both fetal biometry and LMP, similar to the study by Arisouy et al., with nine cases of lymphangioma after prenatal diagnosis. All cases were diagnosed in the second and third trimesters of pregnancy, with a mean gestational age of 22.6 ± 3.9 weeks [16]. In contrast, in the study by Jiao-Ling et al., the mean gestational age of diagnosis of lymphangioma was about 6 weeks later (25 weeks gestation) [14].

Since publications often equate cystic hygroma (lesions appearing in the first trimester of pregnancy) with fetal lymphangioma (lesions appearing in the second half of fetal life), we arbitrarily assumed that only the fetuses with the normal course of the first trimester, e.g., nuchal translucency in these fetuses was normal, will qualify to our test group. It should be highlighted, since most lymphangiomas were found in the region of the neck in midgestation, that our group of fetuses showed normal nuchal translucency when measured in the 1st trimester (12–13th week of gestation).

Cytogenetic test results were available for 50% (*n* = 16) of our patients, which is due to the high rate of fetal deaths before completion of diagnosis (see Table 1). The majority of karyotype results (62.5%, *n* = 10) were normal. Among the abnormal karyotypes, Turner syndrome (25%, *n* = 4) and Down syndrome were observed (12.5%, *n* = 2; see Figure 2). These results are comparable with the available literature, which lists an abnormal karyotype among the risk factors for lymphangioma [17]. Malone, among 134 cases of fetal lymphangioma, found chromosomal anomalies in 51% (*n* = 67) of them, with Down syndrome 18.7% (*n* = 25), Turner syndrome 14.1% (*n* = 19) and Edwards syndrome 13.4% (*n* = 18) being the most common [18]. The differences between the distribution of individual genetic syndromes between our papers may be due to the fact that, in our material, as many as 50% (*n* = 16) of the cases did not receive a genetic diagnosis. In addition, the profile of patients in our department is different because we focus mainly on congenital heart diseases (CHD), which may overestimate the proportion of CHD in the study group.

Some authors have mentioned the coexistence of fetal lymphangioma with other congenital malformations [19]. For example, Malone, among 65 patients diagnosed with separated cystic hygroma without chromosomal abnormalities, found as many as 22 cases of cardiac and skeletal defects, where as many as 8 cases were hypoplastic left heart (HLHS) or right heart syndrome [18]. In addition, both heart defects and lymphangiomas are much more common in patients with Down syndrome and Turner syndrome than in the general population [17,20,21,22]. In our study, 50% of patients with lymphangiomas were diagnosed with CHD; however, these results may be inflated due to the nature of the facility’s operation, i.e., that a large percentage were patients already sent for suspected CHD for a thorough diagnosis. The results cited above indicate that it makes sense to use fetal echocardiography in monitoring the fate of fetuses with lymphangiomas. Up to date, no extensive research has been carried out on this subject. Several echocardiographic examinations in a fetus with lymphangioma allowed for the observation of the lesion and analysis of whether the lymphatic lesion affects cardiac function, e.g., due to compression or flow and cardiac output disturbances [8,23]. In addition, fetal heart echocardiography in the third trimester of pregnancy in a fetus with an extracardiac defect allows us to indicate the time, place, and manner of delivery. This allowed us to determine the optimal management of the fetus and neonate.

In our cases, lymphangioma was predominantly observed in the head and neck regions. In addition, other researchers indicate these locations as the most frequent: head 75% and neck 20% [15]. In Jiao L’s study of 79 fetal lymphangioma cases, lymphangioma was most commonly located in the neck region in 50 fetuses (63.29%) [14], which is consistent with our reports. Rarer locations include other parts of the body, such as: scrotum, retroperitoneum, gluteal region, mediastinum, groin, pelvis, mesentery, omentum and spleen [5,20]. It is difficult to say whether this is related to differences in the Eastern European population compared to Asian population or whether it is simply a coincidence.

The role of prenatal echocardiography in patients with lymphangiomas is not limited to assessing the local effects of the lesion on cardiac structures and blood flow. Lymphangiomas are often accompanied by chromosomal abnormalities, such as Down syndrome and Turner syndrome, which are frequently associated with CHD. Patients with normal karyotype are also relatively more likely to be diagnosed with CHD, especially HLHS. These are poor prognostic factors. Detection and monitoring of CHD and patients with lymphangioma allow for the optimization and prediction of treatment outcomes.

Further studies on this topic should deeply examine the molecular background of lymphangioma, in the meaning of both comparative genomic hybridization (CGH) and copy number variants (CNV).

## 5. Strengths and Limitations

The main advantage of the study is the comprehensive analysis of all prenatal screening data (pregnant women’s age, location, echocardiographic findings, size and location of possible lesion) juxtaposed with the clinical follow-up (used treatment option, treatment effects, survival). We have proposed a new definition of lymphangioma to highlight the differences between this condition and cystic hygroma. It is a novel approach, as it is very common nowadays (or even fashionable) to focus on the 1st trimester, whereas fetal life is very interesting in every aspect and throughout the entire duration of pregnancy. The material includes examinations performed over 21 years at the reference center. Each examination was performed by a physician experienced in prenatal ultrasound/ECHO examination; digital storage was used for offline review [24,25]. Another advantage of the study is the fact of including in the analysis only those fetuses in which the 1st trimester of pregnancy was normal—this approach makes it possible to distinguish lymphangiomas from cystic hygromas, which might possess different embryology backgrounds and, in some cases, may undergo spontaneous regression.

The drawbacks of the current design are its retrospective nature, which prevented the acquisition of fully complete data, e.g., lack of CGH results; karyotype was available in 50% of the analyzed cases (*n* = 16). We presented the data of karyotype that were available during fetal life. Once the baby was born and exhibited no dysmorphic features confirmed by an experienced clinical geneticist, “normal karyotype” was assumed. At the current stage of financial problems in our health system, no further genetic work-up was performed. The prospective nature of the study would have allowed this data to be completed even in cases of miscarriage or termination of pregnancy. Another limitation of the study was the lack of histopathological examination to confirm the lesion and anatomopathological examination in the case of fetal/infant.

## 6. Conclusions

We recommend that fetal lymphangioma should be followed up with echocardiography at a prenatal cardiology referral center, for better prognosis prediction: cases with normal heart anatomy and no functional abnormalities carry a better chance of survival compared to fetuses with lymphangioma and structural or functional heart abnormalities. Moreover, it should be highlighted that, in all lymphangioma cases, there was an indication to perform the karyotype assessment, as there was a high risk of aneuploidy (37%).

## Figures and Tables

**Table 1:** The outcomes of fetuses with lymphangioma considering all cases with isolated lymphangioma as well as fetuses with lymphangioma associated with abnormal ECHO.

The outcomes of all cases	
All cases: Termination of pregnancy 18% (*n* = 6) Death in utero: 44% (*n* = 14) Death after birth: 16% (*n* = 5) Alive: 22% (*n* = 7)	Isolated lymphangioma: Termination of pregnancy: 19% (*n* = 3) Death in utero: 31% (*n* = 5) Death after birth: 19% (*n* = 3) Alive: 31% (*n* = 5)
	Non-isolated lymphangioma: Termination of pregnancy: 19% (*n* = 3) Death in utero: 56% (*n* = 9) Death after birth: 12.5% (*n* = 2) Alive: 12.5% (*n* = 2)
The outcomes of euploid cases	
Euploid cases (*n* = 9) Termination of pregnancy: 44% (*n* = 4) Miscarriage: 11% (*n* = 1) Death after birth: 22% (*n* = 2) Alive: 22% (*n* = 2)	Isolated lymphangioma: Termination of pregnancy: 33% (*n* = 3) Death after birth: 11% (*n* = 1) Alive: 11% (*n* = 1)
	Non-isolated lymphangioma: Termination of pregnancy 11% (*n* = 1) Miscarriage 11% (*n* = 1) Death after birth 11% (*n* = 1) Alive: 11% (*n* = 1)

## Data Availability

Data are available upon request, please contact Iwona Strzelecka (iwona.strzelecka@umed.lodz.pl).

## Supplementary Materials

The following supporting information can be downloaded at: https://www.mdpi.com/article/10.3390/jcm11041035/s1, Table S1: The outcomes of fetuses with lymphangioma based on the localization, CHD, and karyotype.
