# Supplementary material for: The Value of Fetal Heart Evaluation in Fetuses with Rare Congenital Lymphangiomas: A Cohort Study from a Single Tertiary Center across Two Decades (Years 1999–2020)
Source: J Clin Med. 2022 Feb 16;11(4):1035. doi: 10.3390/jcm11041035 (PMC8874571; doi:10.3390/jcm11041035)
Supplement: Supplementary file 1 [file jcm-11-01035-s001.zip › jcm-1574724-supplementary.pdf]

Journal of
Clinical Medicine

MDPI

Supplementary materials

The value of fetal heart evaluation in lymphangioma - rare congenital malformation - in 32 cases from the tertiary center (1999-2020 years)

Paulina Kordjalik *et al.*

Supplementary Table S1. The outcomes of fetuses with lymphangioma based on the localization, CHD, and karyotype.

#	Follow-up	Locations on the body	NHA	CHD	Additional information	Karyotype	Maternal residency in Poland
				**Normal karyotype (n=9)**			
1.	Termination of pregnancy	Left side: cheek, armpit, arm, forearm, behind the ear	+			46,XX	Łódź
2.	Miscarriage	Head, torso, upper limbs, lower limbs	-	DORV,TGA, Pv hypoplasia		46,XX	Inowrocław
3.	Termination of pregnancy	Head, neck, chest	-	DORV, PE		46,XX	Zielona Góra
4.	Termination of pregnancy	Nape	+			46,XX	Łódź
5.	Termination of pregnancy	Nape	+			46,XX	Łódź
6.	Death after birth	Neck	-		Myocarditis	46,XY	No data
7.	Death after birth	Neck	+			46,XY	Radomsko
8.	Alive	Nape, neck	-	VSD,PE		46,XX	Łódź
9.	Alive	Nape	+			46,XY	Włocławek
				**Abormal karyotype (n=6)**			
1.	Miscarriage	Neck, nape	-		SVT	45,X	Voivodeship lodzkie
2.	Miscarriage	Neck	-	AVSD, HAA		45,X	Bydgoszcz
3.	Termination of pregnancy	Neck	-	AVSD, DORV, TOF, VSD		46,XY+21	Płock
4.	Miscarriage	Head, torso, limbs	-	AVSD, DAA		45,X	ND
5.	Alive	Nape	-	AVSD, DORV, PvS, PE		46,XY+21	Poznań
6.	Alive	Nape	+			45,X	ND
				**No data according to karyotype (n=17)**			
1.	Miscarriage	Nape	-	AVSD, A-V v insuff., DV reversal flow		ND	ND
2.	Miscarriage	Neck, nape	-		SVT	ND	Voivodeship lodzkie
3.	Miscarriage	Nape	+			ND	Voivodeship lodzkie

4.	Death after birth	Nape	+			ND	ND
5.	Death in utero	Neck	-	Ebstein s., Pv hypoplasia, TR, MR, Cardiomegaly		ND	Rzeszów
6.	Miscarriage	Nape	-	CHD +		ND	Bełchatów
7.	Death in utero	Neck, nape	-	TGA, PA, TR		ND	ND
8.	Miscarriage	Neck, nape, torso	-	AVSD		ND	ND
9.	Miscarriage	Nape, neck, torso	-		Heart beyond assessment	ND	ND
10.	Death in utero	Nape	-	AVSD, CCHB, Cardiomegaly, TR		ND	ND
11.	Misscariage	Nape	+			ND	Kielce
12.	Death after birth	Neck, from the back of the head to the sacrum	-		CHF	ND	ND
13.	Death after birth	Head, nape	+			ND	ND
14.	Alive	Torso	+			ND	Wrocław
15.	Misscariage	Nape	+			ND	ND
16.	Alive	Nape	+			ND	ND
17.	Alive	Nape	+			ND	ND

Legend: A-V insuff.- Atrial-ventricular insufficiency, AVSD-atrioventricular septal defect, CCHB-complete congenital heart block, CHD-congenital heart defect, CHF-congenital heart failure, DAA-double aortic arch, DORV-double outlet right ventricle, DV- ductus venosus, Ebstein s.-Ebstein syndrome, HAA-aortic arch hypoplasia, MR-mitral regurgitation, NHA-normal heart anatomy, PA-pulmonary atresia, PE-pericardial effusion, PvH-pulmonary valve hypoplasia, Pvhypopl.- pulmonary valve hypo-plasia, PvS- pulmonary valve stenosis, SVT-supraventricular tachycardia, TGA-transposition of great artery, TOF-tetralogy of Fallot, TOP-termination of pregnancy, TR-tricuspid regurgitation, VSD-ventricular septal defect.
